# Tuning the transcription and translation of L-amino acid deaminase in *Escherichia coli* improves α-ketoisocaproate production from L-leucine

**DOI:** 10.1371/journal.pone.0179229

**Published:** 2017-06-29

**Authors:** Yang Song, Jianghua Li, Hyun-dong Shin, Long Liu, Guocheng Du, Jian Chen

**Affiliations:** 1 Key Laboratory of Carbohydrate Chemistry and Biotechnology, Ministry of Education, Jiangnan University, Wuxi, China; 2 Synergetic Innovation of Center of Food Safety and Nutrition, Jiangnan University, Wuxi, China; 3 School of Chemical and Biomolecular Engineering, Georgia Institute of Technology, Atlanta, United States of America; University of Houston, UNITED STATES

## Abstract

α-Ketoisocaproate (KIC) is used widely in the pharmaceutical and nutraceutical industries. In previous studies, we achieved a one-step biosynthesis of KIC from l-leucine, using an *Escherichia coli* whole-cell biocatalyst expressing an l-amino acid deaminase (l-AAD) from *Proteus vulgaris*. Herein, we report the fine-tuning of l-AAD gene expression in *E*. *coli* BL21 (DE3) at the transcriptional and translational levels to improve the KIC titer. By optimizing the plasmid origin with different copy numbers, modulating messenger RNA structure downstream of the initiation codon, and designing the sequences at the ribosome binding site, we increased biocatalyst activity to 31.77%, 24.89%, and 30.20%, respectively, above that achieved with BL21/pet28a-lad. The highest KIC titers reached 76.47 g·L^-1^, 80.29 g·L^-1^, and 81.41 g·L^-1^, respectively. Additionally, the integration of these three engineering strategies achieved an even higher KIC production of 86.55 g·L^-1^ and a higher l-leucine conversion rate of 94.25%. The enzyme-engineering strategies proposed herein may be generally applicable to the construction of other biocatalysts.

## Introduction

α-ketoisocaproate (KIC) is the precursor of the branch-chained amino acid leucine, which can be used as a nitrogen-free substitute for leucine to decrease waste nitrogen accumulation in patients with chronic kidney and hepatic disorders [1]. KIC is also used as a nutraceutical in exercise training to simulate insulin secretion [2], intensify training results [3], and promote muscle recovery after exercise-induced damage [4, 5].

KIC is mainly synthesized via a chemical process that requires the use of toxic reactants that can cause environmental damage [6]. Consequently, the production of KIC via microbial fermentation or biotransformation has attracted attention. For instance, *Corynebacterium glutamicum* was engineered for KIC production and achieved a titer of 9.23 g·L^-1^ [7, 8]. However, the cultivation of this strain depends on expensive nutriment and produces various by-products. In a previous study, we used *Rhodococcus opacus* DSM 43250 to transform l-leucine to KIC with a titer of 1.27 g·L^-1^ [9]. In another recent study, we constructed an *Escherichia coli* BL21(DE3) whole-cell biocatalyst with the membrane-bound l-amino acid deaminase (l-AAD) from *Proteus vulgaris* for the production of KIC from leucine. The highest titer reached 69.1 g·L^-1^ with a leucine conversion rate of 50.3% [10].

The membrane-bound l-AAD from *P*. *vulgaris* is an oxidoreductase that exhibits high substrate specificity. With flavin adenine dinucleotide as a coenzyme, l-AAD catalyzes the deamination of leucine to KIC without generating H_2_O_2_, thereby reducing impacts on host cell growth [11]. Catalysis is proposed to occur via the binding of *Proteus* l -AADs to the electron transport chain to reduce O_2_ to H_2_O [12]. Furthermore, structural analysis shows that l-AAD has a transmembrane peptide at the N-terminus and an extracellular C-terminus that prevent other intracellular metabolic reactions owing to the membrane barrier [13]. However, low l-AAD expression limits KIC production. Therefore, we performed optimizations at the transcriptional and translational levels of l-AAD in the present study. Many strategies are available for improving enzyme activity, including directed evolution, plasmid selection, transcription control, and translation modulation [14, 15].

In this study, we attempted to increase L-AAD activity by tuning transcriptional and translational levels to further improve KIC titer. The N-terminal codon of l-AAD in the downstream region was initially redesigned, and then the ribosome-binding site (RBS) sequences were modulated to improve translation efficiency. In addition, the plasmid origin was optimized to offer various amounts of the DNA template for transcription. Finally, these three strategies were combined to improve the expression levels of l-AAD, and the KIC titer and leucine conversion ratio reached 86.55 g/L and 94.25%, respectively.

## Materials and methods

### Software

To predict changes at the N-terminus of l-AAD, we used the software Mfold to predict messenger RNA (mRNA) structure and thermodynamic free energy [16]. For the optimization of RBS sequences, RBS Calculator v2.0 (https://salislab.net/software/forward) was used to evaluate expression levels [17]. We optimized the RBS sequences by choosing 27 synthetic RBS sequences with ΔG_tot_ values ranging from –4.67 to 12.35 kcal·mol^-1^ (S1 Table).

### Chemicals, strains, and plasmids

*E*. *coli* BL21 (DE3) carrying the gene l-AAD from *P*. *vulgaris* was used as the control strain [10]. For the optimization of plasmid copy number, strains were constructed by inserting the l-AAD gene in the *Nco*I and *Xho*I restriction enzyme sites of plasmids pColADuet-1, pACYCDuet-1, pRSFDuet-1, pETDuet-1, and pCDFDuet-1. The mutations at the N-terminal codon were introduced with a QuickChange Site-Directed Mutagenesis Kit (from TaKaRa Bio Inc., Dalian, China), and the RBS mutants were generated using a MutanBEST kit (from TaKaRa Bio Inc., Dalian, China). All mutant sequences are given in S1 and S2 Tables. The strains and plasmids used in the study are listed in Table 1, and the oligonucleotides appear in S3 Table. Restriction enzymes, blunting enzymes, T4 DNA ligase, DNA purification kits, Plasmid DNA MiniPreps kits, and MutanBEST kits were purchased from TaKaRa (Dalian, China). Standard KIC was purchased from Sigma-Aldrich (St. Louis, MO, USA).

**Table 1:** The strain and plasmid used in this study.

Plasmids/strains	Description	Source or reference
**pET28a**	T7 promoters, pBR322 ori, Kn^R^	Novagen
**pRSFDuet-1**	Double T7 promoters, RSF ori, Kn^R^	Novagen
**pETDuet-1**	Double T7 promoters, pBR322 ori, Amp^R^	Novagen
**pCDFDuet-1**	Double T7 promoters, CloDF13 ori, Sm^R^	Novagen
**pACYCDuet**	Double T7 promoters, p15A ori, Cm^R^	Novagen
**pColADuet-1**	Double T7 promoters, ColA ori, Kn^R^	Novagen
**BL21/pET28a-lad**	pET28a carrying L-AAD gene	(10)
**BL21/pRSFDuet-lad**	pRSFDuet-1 carrying L-AAD gene	This study
**BL21/pETDuet-lad**	pETDuet-1 carrying L-AAD gene	This study
**BL21/pCDFDuet-lad**	pCDFDuet-1 carrying L-AAD gene	This study
**BL21/pACYCDuet-lad**	pACYCDuet-1 carrying L-AAD gene	This study
**BL21/pColADuet-lad**	pColADuet-1 carrying L-AAD gene	This study
**BL21/ RBS1~27**	pET28a carrying RBS mutants	This study
**BL21/A2/S4/R5/R6/I9/I10/G11**	pET28a carrying synonymy codon mutant	This study
**BL21/I3Δ/S4Δ/R5Δ/K7Δ/F8Δ**	pET28a carrying a codon deletion	This study
**BL21/pACYCDuet-A2**	pACYCDuet-1 carrying the A2 mutant and L-AAD gene	This study
**BL21/pACYCDuet-RBS6**	pACYCDuet-1 carrying the RBS6 mutant and L-AAD gene	This study
**BL21/A2-RBS6**	pET28a carrying the RBS6 mutant, A2 mutant and L-AAD gene	This study
**BL21/pACYCDuet-A2-RBS6**	pACYCDuet-1 carrying the RBS6 mutant, A2 mutant and L-AAD gene	This study

### Whole-cell biocatalyst preparation

The recombined strains were cultured in 25 mL Luria-Bertani medium with corresponding antibiotics at 37°C overnight. The final concentrations of antibiotics were 20 μg/mL chloramphenicol, 40 μg/mL kanamycin, 40 μg/mL streptomycin, and 100 μg/mL ampicillin. The seed cultures (2%, v/v) were transferred into Terrific broth medium with corresponding antibiotics and cultured at 37°C and 200 rpm. Isopropyl-beta-d-thiogalactopyranoside (final concentration, 0.4 mM) was added when the optical density at 600 nm reached 0.6, and expression was induced for 4 h at 37°C and 200 rpm. Then, the cells were collected via centrifugation at 10,000 × *g* for 5 min at 4°C. The cells were washed twice with phosphate buffer (pH 7.5) before use. The optical density at 600 nm was measured with a spectrophotometer (UV-2450 PC, Shimadzu Co., Kyoto, Japan).

### Cell density measurement and growth rate analysis

To determine the dry cell weight (DCW; g·L^-1^), we dried 20 mL of cells and divided the weight (grams) by 20 mL. For the measurement of cell growth rate, seeds (2%, v/v) were added to TB medium with antibiotics and divided into 96-well plates in eight replicates. Then, the absorbance at 600 nm was measured hourly until the stationary phase. The data were analyzed with OriginPro 8.5 to obtain the maximum specific growth rate (*μ*_max_).

### Assay of biocatalyst activity and leucine feeding strategy

Biocatalyst activity was measured by mixing prepared cells and 1 mL leucine solution (100 mM leucine, pH 7.5) with a final cell density 0.8 g·L^-1^ in 5-mL centrifugation tubes at 37°C for 30 min. Then, the reaction was stopped with centrifugation at 10,000 × *g* for 5 min. The KIC concentration in the supernatant was measured by HPLC. The biocatalyst activity was calculated using a previously reported equation (Eq 1) [10]:

$$v=\frac{C\;(KIC)}{DCW\times T}$$ (1)

where *ν* is the catalyst activity (mg·L^-1^·min^-1^·g^-1^ DCW), *C* (KIC) is the concentration of KIC (mg·L^-1^), DCW is the dry cell weight (g·L^-1^), and *T* is the reaction time (30 min).

For KIC production, cells (0.8 g·L^-1^) were resuspended in 20 mL leucine solution (100 mM, pH 7.5) in 250-mL flasks. Then, 13.1 g/L leucine powder was added into the reaction solution every 2 h (total of six additions). A sample was obtained every 4 h, and KIC concentration was measured as described below.

### Sodium dodecyl sulfate-polyacrylamide gel electrophoresis (SDS-PAGE) analysis

The cells collected in the previous step were lysed via ultrasonication, and the samples were prepared in denaturing buffer at 70°C for 10 min. Fifteen microliters of sample was loaded onto a gel containing 10% polyacrylamide separating gel (Invitrogen, CA, USA). The marker purchased from TransGen (Beijing, China) and SeeBlue Plues2 (Invitrogen, CA, USA).

### RNA preparation and quantitative reverse transcription polymerase chain reaction (qRT-PCR)

qRT-PCR was performed to evaluate gene transcription levels. The strains (with different origins) were harvested after a 1-h induction and immediately frozen in liquid nitrogen to avoid mRNA changes. The mRNA was extracted with an RNAisoPlus kit (TaKaRa, Dalian, Japan). Complementary DNA was generated with a PrimeScript RT Reagent Kit Perfect Real Time (Takara, Otsu, Japan). The qRT-PCR parameters were selected according to a previous study [18], and the primers are shown in S3 Table. The transcript of a 16s ribosomal RNA processing protein (rimM) was the reference for internal control. All reactions were performed in triplicate with three biological replicates.

### HPLC analysis of KIC concentration

The KIC concentration in the supernatant was measured with HPLC (Agilent 1200 series, Santa Clara, USA) using an Agilent ZORBAX SB-Aq column (4.6 × 250 mm, 5 μm). The mobile phase contained a mixture of 10% methanol and 90% diammonium phosphate (pH 2.50) at a flow rate of 0.6 mL/min. The column temperature was 35°C, and the injection volume was 10 μL. KIC concentration was detected with an ultraviolet detector at a wavelength of 203 nm [10].

## Results

### Effect of l-AAD N-terminal codon modulation on biocatalyst activity and KIC production

The l-AAD translation initiation region is a critical effector of gene expression. It contains an untranslated region and a 5’ mRNA-coding sequence [19]. The codon at the N-terminus also has a strong effect on gene expression. Designing N-terminal codons by changing the secondary structure of mRNA has been shown to affect initial ribosomal elongation and increase translational efficiency [20]. Bias in the codon can further change the mRNA structure, and this alteration is tightly related to protein expression [21]. Therefore, we used two strategies to design the mRNA structure to improve the l-AAD expression level—namely, we designed a series of N-terminal codon substitutions based on codon bias and deleted the loop of mRNA in the hairpin to change the mRNA structure.

**Fig 1:**
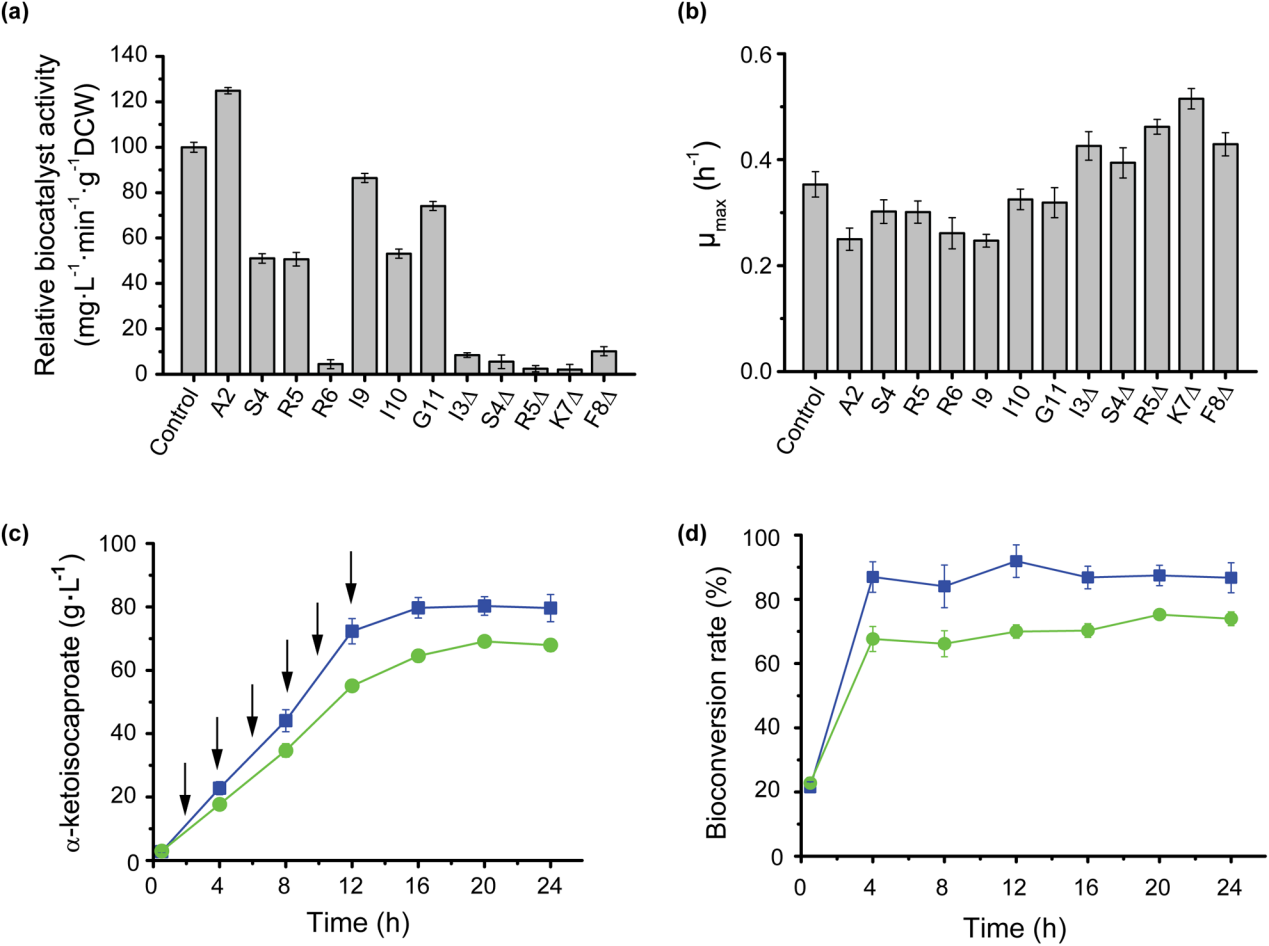
The effect of mutants in 5’-gene sequences on L-AAD catalyst activity, cell growth and KIC production. **a** and **b**: The effect of mutant at 5’-gene sequences on biocatalyst activity and cell growth, respectively. **c** and **d**: The comparison of KIC production and bioconversion of BL21/A2 and (blue line) and BL21/pET28a-lad (green line). The arrows showed the additon of L-leucine.

The modified mRNA was introduced via site-directed mutagenesis, and the theoretical translation efficiency was designed using the calculated minimal folding free energy (ΔG; ranging from –2.0 to –6.1 kcal·mol^-1^), which reflected the stability of the mRNA secondary structure. The mutants produced through N-terminus modification are listed in the S1 Table. These mutants changed biocatalyst activity from 2.01% (K7Δ) to 124.89% (A2) compare with that of the control (Fig 1A). The results reflected a difference between the theoretical translation value and the actual enzyme activity observed with these two strategies. The biocatalyst activity increased as ΔG increased in the codon bias mutants. However, the deletion of the codon in the hairpin loop yielded biocatalyst activities that were 10% below that of the control, which indicated that these deletion mutants significantly influenced catalytic efficiency (Fig 1A). One explanation for these results may be the failure of protein expression due to the deletion of some amino acids in the signal peptide [22]. Previous studies showed that l-AAD expression influences cell growth, which is crucial in preparing whole-cell biocatalysts [13]. In the present study, the cell-specific growth rate decreased when biocatalyst activity increased (Fig 1B).

Fig 1C shows the KIC titer of the mutant and control strains. The highest KIC titer of BL21/A2 reached 80.29 g·L^-1^ with a leucine conversion rate of 87.43%, an increase of 16.19% over the yield and conversion of the control strain. Fig 1D shows the bioconversion value of the substrate. During the first phase (0–0.5 h), bioconversion was low owing to the excessive substrate level in the solution. During the next 12 h, 100 mM substrate once was fed into the culture at 2-h intervals, and the bioconversion rate was relatively stable owing to the balance of biotransformation and substrate supplement. After 12 h, no substrate was added, and the bioconversion rate changed slowly, which indicated that enzyme thermostability and product inhibition limited increases in KIC production. The supplied strategies have been optimized in our previous study with the consideration of biocatalyst activity and supply intervals [10].

### Effect of RBS changes on l-AAD expression and KIC production

**Fig 2:**
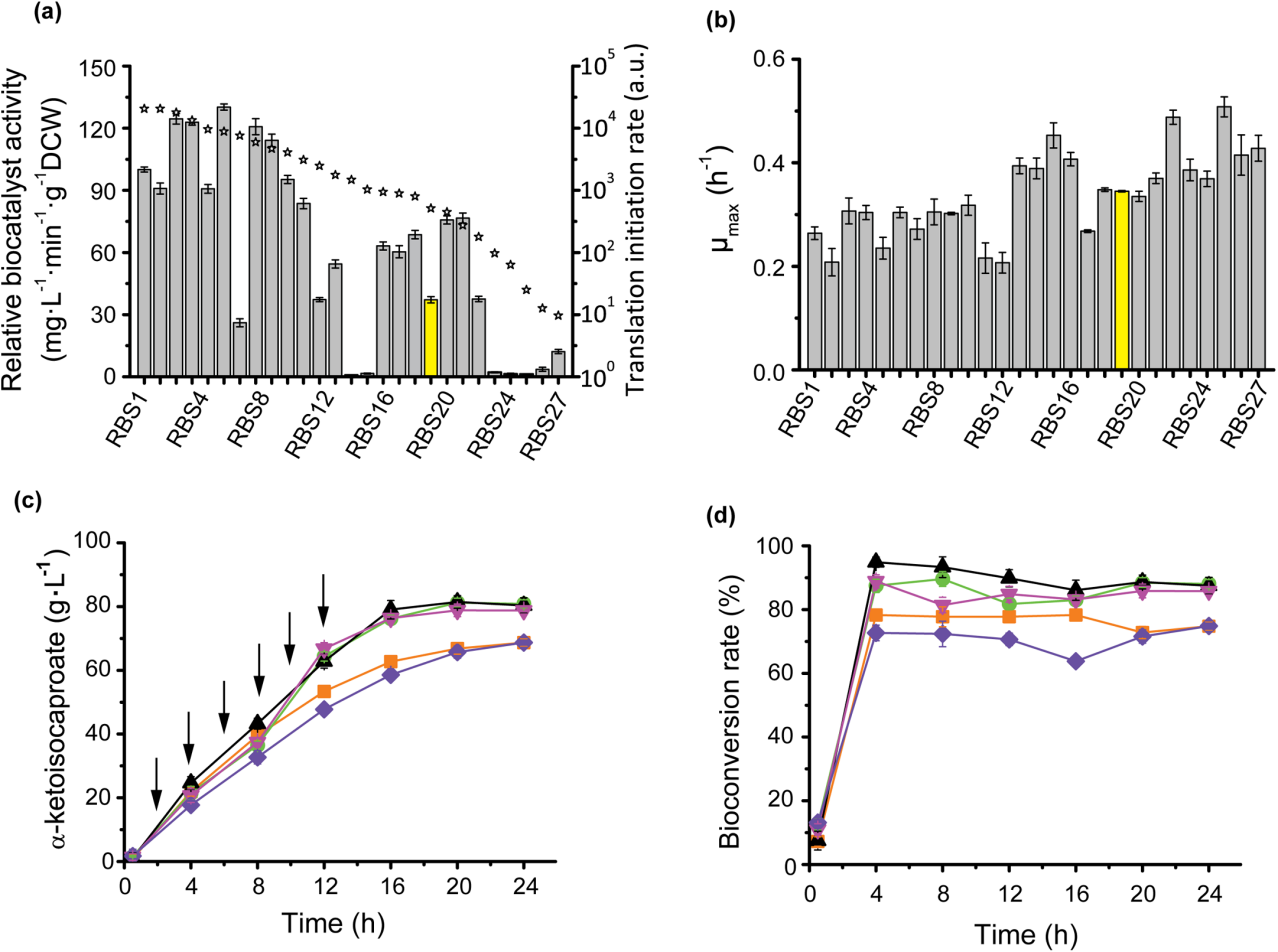
The effect of ribosome binding site design on L-AAD catalyst activity, cell growth and KIC production. **a**: The comparison of biocatalyst activity with predicted translation rate. Gray bar: the biocatalyst activity and the BL21/pET28a-lad as the control (the yellow bar). ☆: predicted translation initiation rate. **b**: The effect of RBS mutants on cell growth. **c** and **d**: the KIC production and bioconversion rate of the 4 dominant RBS mutants and BL21/pET28a-lad. BL21/RBS3 (orange squares), BL21/RBS4 (green circles), BL21/RBS6 (black triangles), BL21/RBS8 (magenta triangles), BL21/pET28a-lad (violet rhombus), the additon of L-leucine (arrows).

In prokaryotes, translation initiation efficiency is the rate-limiting step in translation process. The translation initiation process included multiple molecular identification and combination, such as the identification of 16S rRNA to RBS site, the combination of tRNA^fMET^ to ATG codon, the space between RBS to the start codon and the mRNA secondary structure. Therefore, the RBS sequences control the identification of 16S rRNA to RBS sequence and mRNA secondary structure directly, and affect the binding of tRNA^fMET^ to ATG codon indirectly, resulting in tune protein expression [23]. We designed a series of synthetic RBS sequences with an RBS calculator to predict l-AAD translation levels [17]. So the accessibility of ribosomes to mRNA (ΔG_tot_) and resulting expression levels were predicted with the known thermodynamic model [14], which yielded a library with a 2126-fold translation initiation rate range and 27 synthetic RBS sequences with ΔG_tot_ values ranging from –4.67 to 12.35 kcal·mol^-1^ were chosen in this study (S2 Table). The biocatalyst activity of the whole cell showed the trends predicted by the thermodynamic model (Fig 2A). Among the RBS mutants, BL21/RBS3, BL21/RBS4, BL21/RBS6, and BL21/RBS8 biocatalyst activity increased by 24.52%, 22.86%, 30.20%, and 20.74%, respectively, above that of the wild-type RBS strain. The maximum cell-specific growth rate was negatively related to biocatalyst activity, which proved that l-AAD expression has a negative effect on *E*. *coli* growth (Fig 2B). The BL21/RBS6 mutant produced the highest KIC titer of 81.41 g·L^-1^ with a leucine conversion rate of 88.66% (Fig 2C and 2D). The bioconversion rate showed the same trend as that in Fig 1D owing to the use of the same substrate feeding strategy.

### Effect of changing plasmid copy number on biocatalyst activity, cell growth, and KIC production

**Fig 3:**
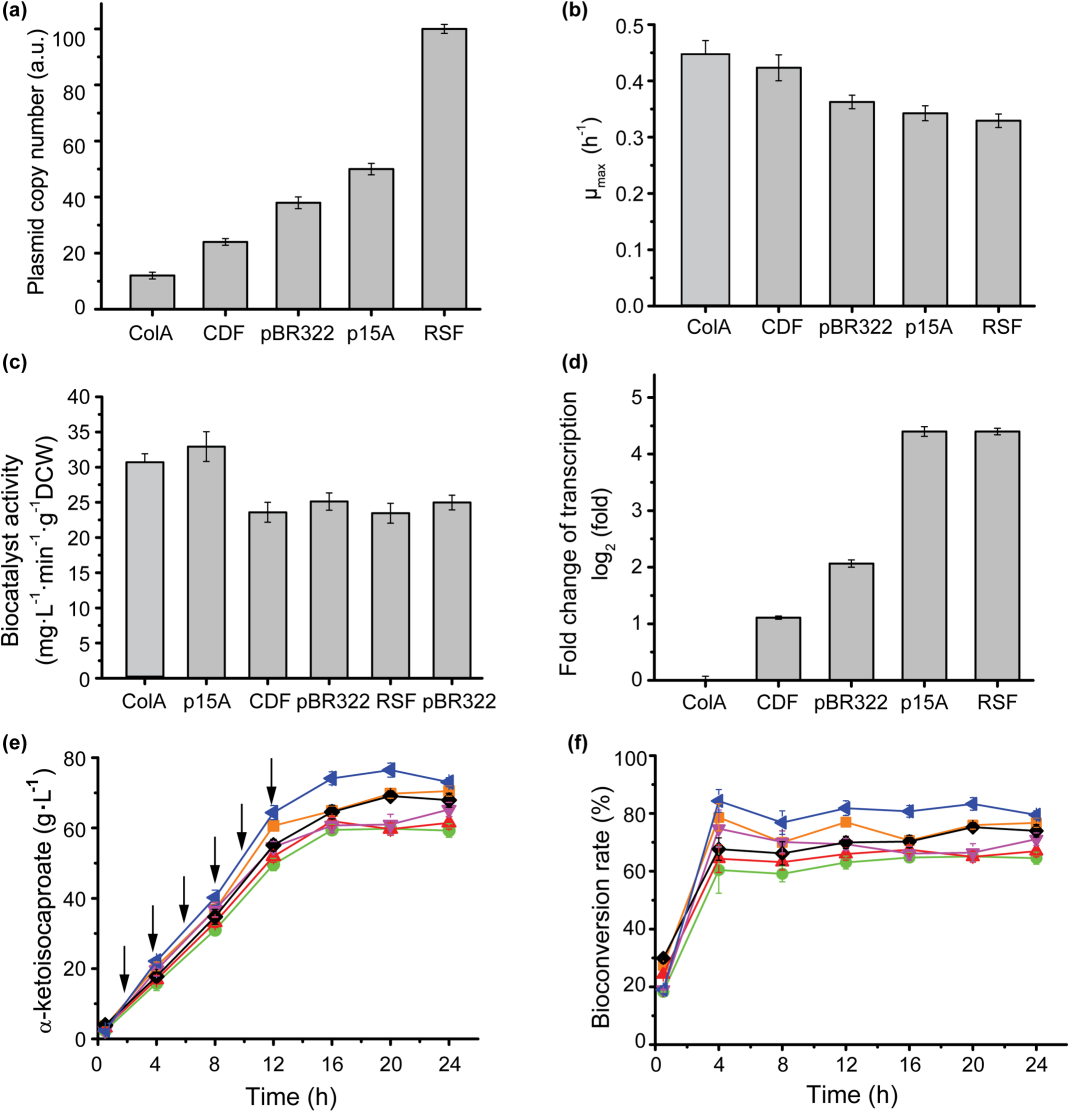
The effect of different origin on cell growth, biocatalyst activity, transcriptional level and KIC production. **a**: the actual plasmid copy number under different origin. **b**: The effect of different plasmid origin on cell growth. **c**: The effect of different origin on biocatalyst activity. **d**: The qRT-PCR of L-AAD in different origin strains. **e**: The α-ketoisocaproate production of different origin strains **f:** The bioconversion rate of different origin strains. ColA (orange squares), p15A (blue triangles), CDF (green circles), pBR322 (red triangles), RSF (magenta triangles), pBR322* from BL21/pET28a-lad (black rhombus), the additon of L-leucine (arrows).

Plasmids are a clutter of DNA elements that can affect host growth according to copy number and the properties of delivered genes [24]. Changes in plasmid copy number are often used to control pathway flux in metabolic engineering [25]. In this study, the transcription level was regulated by different plasmid origins that offered different template to transcript. pColADuet-1 (ColA origin), pCDFDuet-1 (CDF origin), pETDuet-1 (pBR322 origin), pACYCDuet-1 (p15A origin), and pRSFDuet-1 (RSF origin) have the same plasmid backbone and the same transcript initiation site (T7 promoter), so l-AAD expression levels (l-AAD from *P*. *vulgaris*) were regulated by different plasmid origins. The plasmid copy number of pColADuet-lad (ColA origin), pCDFDuet-lad (CDF origin), pETDuet-lad (pBR322 origin), pACYCDuet-lad (p15A origin), and pRSFDuet-lad (RSF origin) were measured and calculated to be 12, 24,38, 50, and 100, respectively (Fig 3A). As expected, the excess plasmid copy number increased the metabolic load, so the maximum specific growth rate decreased as plasmid copy number increased (Fig 3B) [26], which led to increase of the production cost in whole-cell biocatalyst preparation. However, the biocatalyst activity increased consistently with the increase of plasmid copy number. BL21/pACYCDuet-lad (p15A), which had a relative high copy number had the highest biocatalyst activity of 32 mg·L^-1^·min^-1^·g^-1^ DCW (Fig 3C), but the higher copy number (RSF origin) did not have the higher biocatalyst activity.

To determine the effect of plasmid copy number on transcription, we examined l-AAD mRNA levels. The mRNA levels increased with the augment of plasmid copy number (Fig 3D), which suggests that transcription is regulated by plasmid copy number. The BL21/pACYCDuet-lad (p15A) strain produced the highest KIC titer of 76.47 g·L^-1^ at 20 h (Fig 3E). We also studied the effects of plasmid copy number on the bioconversion rate and found that the rate was stable with substrate feeding (Fig 3F), as described in the previous two sections.

### Integration of the three strategies

**Fig 4:**
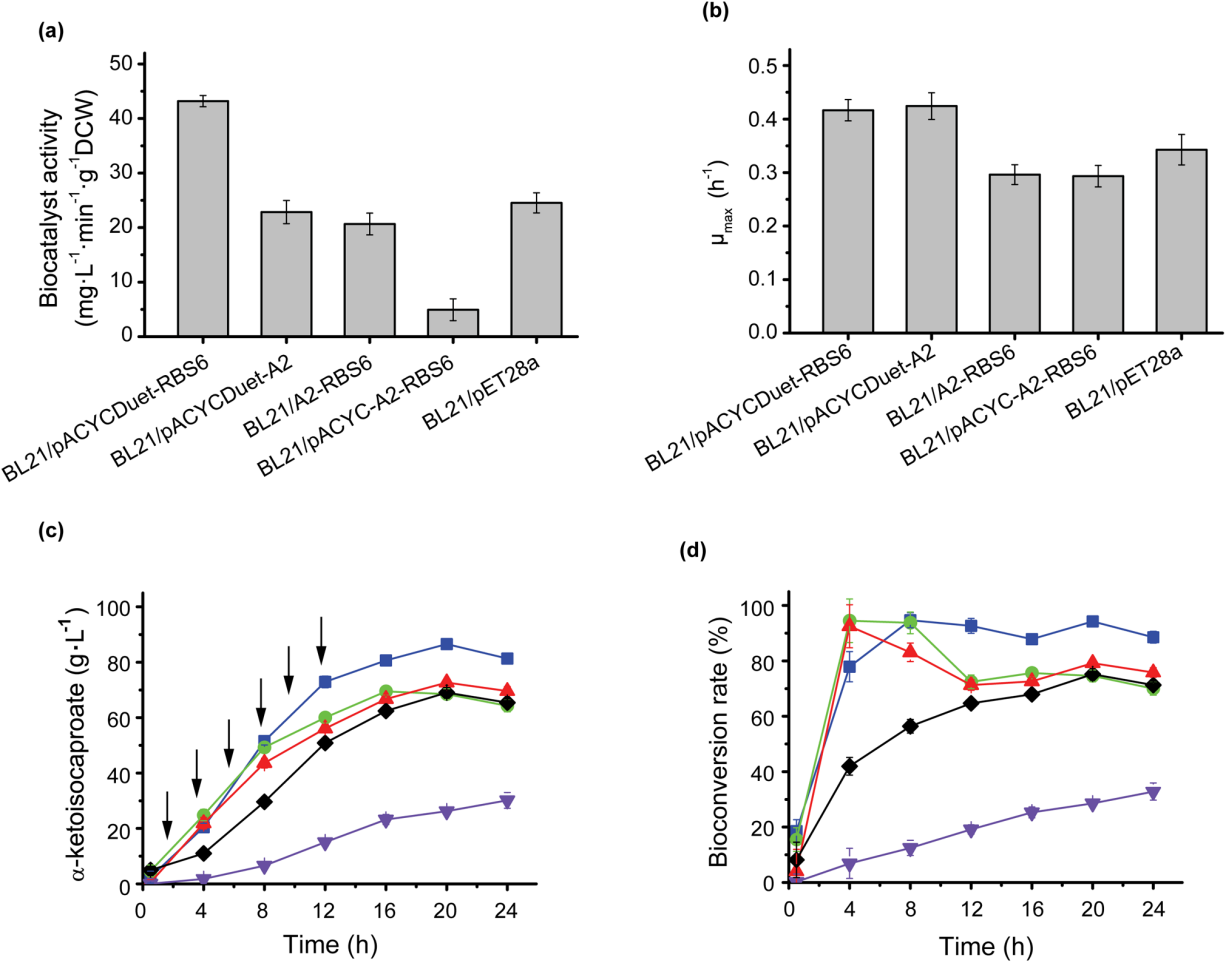
The effect of integration of dominant conditions at transcription and translation. **a**: the effect of integration of dominant conditions on biocatalyst activity. **b**: the effect of integration of dominant conditions on cell growth. **c** and **d**: The KIC production and bioconversion rate of integrated strains and BL21/pET28a-lad. BL21/ACYCDuet-RBS6 (blue squares), BL21/ACYCDuet-A2 (green circles), BL21/A2-RBS6 (red triangles), BL21/ACYC-A2-RBS6 (violet triangles), BL21/pET28a-lad (black rhombus), the additon of L-leucine (arrows).

In response to the results obtained with the three regulation strategies described above, we combined these approaches in an attempt to increase l-AAD expression levels and KIC titers further. We assembled the optimal conditions in pairs to generate three combined mutants (BL21/pACYCDuet-A2, BL21/pACYCDuet-RBS6, and BL21/A2-RBS6) and generated one mutant that combined all three optimal conditions (BL21/pACYCDuet-A2-RBS6). Fig 4A shows that the integration of optimal RBS conditions and plasmid copy number in BL21/pACYCDuet-RBS6 increased biocatalyst activity to levels to 31.20% and 51.50% above those obtained with single-condition optimization in BL21/pACTCDuet-LAD and BL21/RBS6, respectively. However, the integration of two translation conditions had a negative effect on biocatalyst activity (BL21/A2-RBS6 and BL21/pACYCDuet-A2-RBS6). The μ_max_ values also fit biocatalyst activity inversely (Fig 4B). KIC production with added leucine further increased to 86.55 g·L^-1^ with a bioconversion rate of 94.25% (Fig 4C). The HPLC chromatograms for the highest production and the control is shown in S1 Fig. In addition, the strains with the mutant at A2 (BL21/ACYCDuet-A2 and BL21/A2-RBS6) had higher bioconversion rates during first 8 h, whereas BL21/pACYCDuet-RBS6 had the highest bioconversion rate after 12 h (Fig 4D).

## Discussion

In a previous study, we achieved one-step synthesis of KIC by constructing an *E*. *coli* whole-cell biocatalyst expressing l-AAD from *P*. *vulgaris*. KIC titers reached 69.1 g·L^-1^ in a fed-batch process with a leucine conversion rate of 50.02% [10]. However, this process was not highly attractive in terms of KIC production and bioconversion rate. To improve KIC titers, we designed three approaches to increase the biocatalyst activity at the transcriptional and translational levels. These strategies increased enzyme expression. Thus, maximum KIC production reached 86.55 g·L-1 with a bioconversion rate of 94.25%. The HPLC graph in S1 Fig showed the result. These results demonstrate the effectiveness of enhancing l-AAD expression.

**Fig 5:**
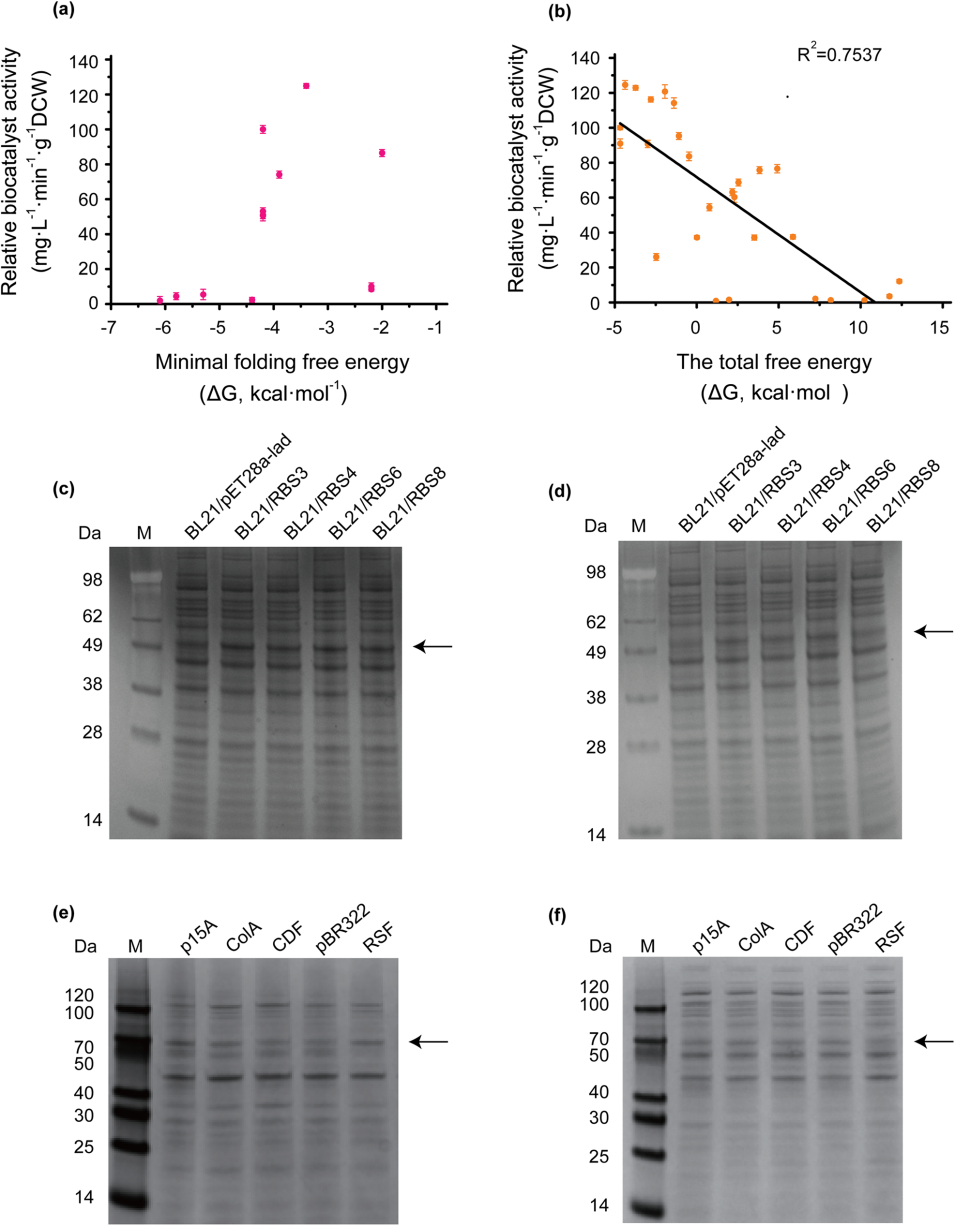
The reliability of tune translation level strategies and the SDS-PAGE analysis of protein expression. **a**: For 5’-gene sequence mutants, the biocatalyst activity was compared with minimal folding free energy (ΔG) value. **b**: For RBS mutants, the biocatalyst activity was compared with the total free energy ΔG_tot_. **c:** SDS-PAGE analysis of total cell lysates of recombinant *E*. *coli* with different RBS sequences. **d**: SDS-PAGE analysis of cell lysate supernatant of recombinant *E*. *coli* with different RBS sequences. **e:** SDS-PAGE analysis of total cell lysates of recombinant *E*. *coli* at different plasmid copy number. **f**: SDS-PAGE analysis of cell lysate supernatant of recombinant *E*. *coli* at different plasmid copy number. Band indicated by arrow.

In the translational approach, the translation initiation efficiency was affected by mRNA structure and fold energy, and the untranslated and downstream regions in particular played crucial roles in reliable gene expression [15]. Therefore, strategies to increase the thermodynamic free energy barrier of mRNA were introduced in this study, including codon changes and changes in mRNA secondary structure. However, for practical secretion expression procedures, the achievement of l-AAD biocatalyst activity should be considered a complex process including protein folding, other translational regulation element and protein secretion limitation. Therefore, the mRNA minimal folding energy (ΔG), which is a factor affecting the translation initiation efficiency, does not have a linearity relation with the biocatalyst activity (Fig 5A). A relative 124.89% biocatalyst activity at a proper ΔG (–3.4 kcal·mol^-1^) was obtained in this study.

Therefore, a more precise modulation is applied to tune translation level, in which the thermodynamic model is based on an equation that considers the hybridization of mRNA and ribosomal RNA, improper spacing, the hybridization of start codon and transfer RNA, mRNA fold energy, and the energy required to maintain the unfolded mRNA structure [14]. RBS modulation was the most powerful approach to the tuning of translation initiation efficiency, because it most effectively tuned protein expression, thereby achieving results that closely matched those predicted by the model. Fig 5B shows that biocatalyst activity changed depending on ΔG_tot_ (R^2^ = 0.7537). However, the biocatalyst activity could not reflect the protein expression directly, the expression level of the four high biocatalyst activity mutants (BL21/RBS3, BL21/RBS4, BL21/RBS6, and BL21/RBS8) was analyzed by SDS-PAGE. The L-AAD is linked to the membrane by a peptide and the active center is out of the membrane [27]. So the total cell lysates included the inclusion bodies and active enzyme, but the cell lysate supernatant is just active enzyme because the membrane could not be separated from supernatant by such a low speed centrifugation. The RBS mutants have a relative high protein expression level than the wild-type in both the total cell lysates and cell lysate supernatant (Fig 5C and 5D), which suggested the improving of the translation initiation led to more protein in both inactive and active form.

In the transcriptional regulation, the copy numbers of the ColA, CDF, pBR322, p15A and RSF plasmid origins were 12, 24,38, 50, and 100, respectively [28]. The biocatalyst activity and mRNA levels increased as expected. To avoid the influence of other factors such as insoluble proteins, inclusion bodies, and protein transportation involved in the enzyme expression process, we performed sodium dodecyl sulfate-polyacrylamide gel electrophoresis analysis of the total cell lysates and cell lysate supernatant to assess active protein expression (Fig 5E and 5F). The increase of plasmid copy number resulted in total cell lysates and the expression levels of l-AAD increased significantly (except RSF). The highest plasmid copy number (RSF origin) have a high mRNA level (as same as p15A origin) and relative high total protein (a little less than p15A origin), but do not have a high active protein, which indicated that the excessive high plasmids yielded more inactive proteins. The high plasmid copy number will increase the metabolic load of strain, and the enzyme expression is also regulated by other factors such as protein folding, transportation and so on [26]. Therefore, the appropriate plasmid copy numbers and mRNA structure regulations are significant effectors of protein expression and the bioconversion process. The highest biocatalyst activity was observed with the p15A origin at a relative high copy number. Even in metabolic engineering aimed at controlling the lycopene biosynthesis pathway, the plasmids with mid-level copy numbers achieved an approximate 2-fold greater target production compared with that of high-copy-number plasmids [29].

Beyond the regulation of transcription and translation, the kinetic model and known regulated elements convinced us to design for quantitative regulation within sequences and expression, but enzyme activity may be affected by other factors that cannot be understood by simply expressing a fluorescence protein as used in many other studies [20]. Notably, l-AAD is a transmembrane protein, deaminase activity also appears to rely not only on the gene transcription and translation but also on correct folding and transport as well as the metabolic load of the strain. However, design at the transcriptional and translational levels effectively improves biocatalyst activity and KIC titers.

### Statistical analysis

All experiments were performed at least three times, and the results were expressed as means ± standard deviation (n = 3).

## Supporting information

S1 Fig The HPLC chromatograms for the highest production and the control. (a) The standard sample. KIC 1 g/L. (b) The control production 69.06 g/L, with diluted 50 times. (c) The highest production 85.55 g/L, with diluted 50 times.
(TIF)

S1 Table The ΔG and sequences at N-terminal modification. (DOCX)

S2 Table The sequence, translation initiation rate and ΔGtot of the RBS mutants. (DOCX)

S3 Table Primers used in the study. (DOCX)
