# Supplementary figures and images for: Tuning the transcription and translation of L-amino acid deaminase in Escherichia coli improves α-ketoisocaproate production from L-leucine
Source: PLoS One. 2017 Jun 29;12(6):e0179229. doi: 10.1371/journal.pone.0179229 (PMC5491005; doi:10.1371/journal.pone.0179229)

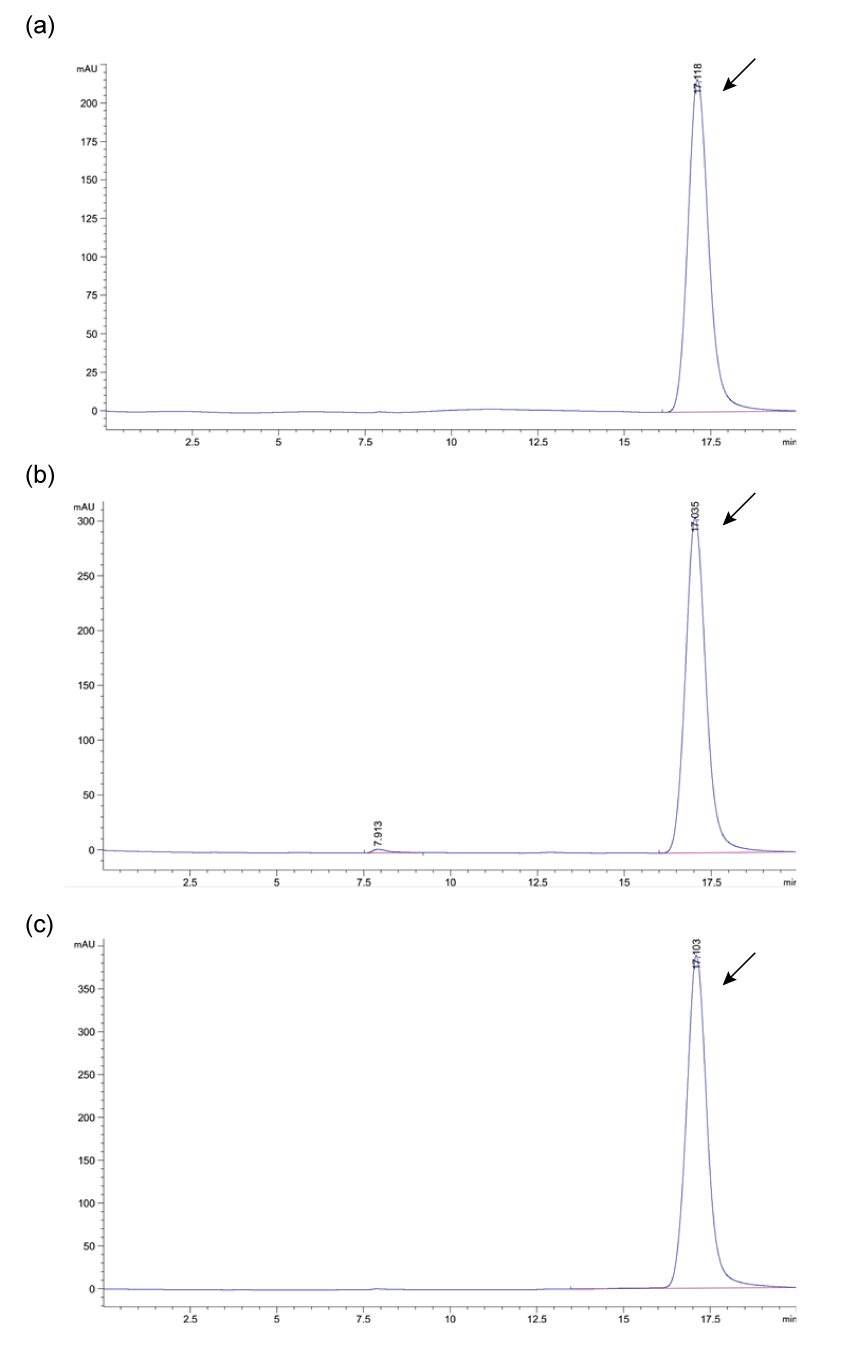

Supplement: S1 Fig — (a) The standard sample. KIC 1 g/L. (b) The control production 69.06 g/L, with diluted 50 times. (c) The highest production 85.55 g/L, with diluted 50 times. (TIF) [file pone.0179229.s001.tif]
