# Supplementary material for: Tuning the transcription and translation of L-amino acid deaminase in Escherichia coli improves α-ketoisocaproate production from L-leucine
Source: PLoS One. 2017 Jun 29;12(6):e0179229. doi: 10.1371/journal.pone.0179229 (PMC5491005; doi:10.1371/journal.pone.0179229)
Supplement: S1 Table — (DOCX) [file pone.0179229.s002.docx]

**S1 Table. The ΔG and sequences at N-terminal modification.**

| Name | ΔG (Kcal/mol) | The sequences at the N-terminal region |
| --- | --- | --- |
| wild | -4.2 | atggcgatatctagaagaaaatttatcattggt |
| A2 | -3.4 | atg**gcT**atatctagaagaaaatttatcattggt |
| S4 | -4.2 | atggcgata**tcA**gaagaaaatttatcattggt |
| R5 | -4.2 | atggcgatatct**agG**agaaaatttatcattggt |
| R6 | -5.8 | atggcgatatctaga**agG**aaatttatcattggt |
| I9 | -2.0 | atggcgatatctagaagaaaattt**atA**attggt |
| I10 | -4.2 | atggcgatatctagaagaaaatttatc**atA**ggt |
| G11 | -3.9 | atggcgatatctagaagaaaatttatcatt**ggA** |
| I3Δ | -2.2 | atggcg---tctagaagaaaatttatcattggt |
| S4Δ | -5.3 | atggcgata---agaagaaaatttatcattggt |
| R5Δ | -4.4 | atggcgatatct---agaaaatttatcattggt |
| K7Δ | -6.1 | atggcgatatctagaaga---tttatcattggt |
| F8Δ | -2.20 | atggcgatatctagaagaaaa---atcattggt |
