# Supplementary material for: Tuning the transcription and translation of L-amino acid deaminase in Escherichia coli improves α-ketoisocaproate production from L-leucine
Source: PLoS One. 2017 Jun 29;12(6):e0179229. doi: 10.1371/journal.pone.0179229 (PMC5491005; doi:10.1371/journal.pone.0179229)
Supplement: S2 Table — (DOCX) [file pone.0179229.s003.docx]

**S2 Table. The sequence, translation initiation rate and ΔGtot of the RBS mutants.**

| Name | RBS Sequence | Translation Initiation Rate (a.u.) | Δ G_total_ (Kcal/mol) |
| --- | --- | --- | --- |
| RBS1 | TACGGT | 20496.44 | -4.67 |
| RBS2 | TGCGGT | 20496.44 | -4.67 |
| RBS3 | CTGCGG | 17864.95 | -4.37 |
| RBS4 | CTACGG | 13394.07 | -3.73 |
| RBS5 | GCGGTA | 9588.6 | -2.99 |
| RBS6 | CGGTTA | 8826.57 | -2.8 |
| RBS7 | TACGGA | 7615.24 | -2.47 |
| RBS8 | TATGGT | 5972.27 | -1.94 |
| RBS9 | GCGGCG | 4666.94 | -1.39 |
| RBS10 | TCGGTC | 4077.52 | -1.09 |
| RBS11 | GGGCGG | 3088.44 | -0.47 |
| RBS12 | ACGGGT | 2485.42 | 0.01 |
| RBS13 | GCCCGG | 1767.58 | 0.77 |
| RBS14 | CCCGGC | 1479.92 | 1.17 |
| RBS15 | CTATGT | 1030 | 1.97 |
| RBS16 | CTTGGT | 943.6 | 2.17 |
| RBS17 | GTAGTA | 890.78 | 2.29 |
| RBS18 | TGGTAG | 798.14 | 2.54 |
| **RBS19 (wild-type)** | **AAGGAG** | **514.42** | **3.51** |
| RBS20 | CTCGAC | 447.03 | 3.83 |
| RBS21 | ACTTCT | 273.96 | 4.91 |
| RBS22 | CCGAGG | 178.06 | 5.87 |
| RBS23 | CACGCC | 96.78 | 7.23 |
| RBS24 | CCATGT | 63.25 | 8.17 |
| RBS25 | TTCTAG | 25.03 | 10.23 |
| RBS26 | AGAGCC | 12.63 | 11.75 |
| RBS27 | ATAGCC | 9.64 | 12.35 |
