# Supplementary material for: Tuning the transcription and translation of L-amino acid deaminase in Escherichia coli improves α-ketoisocaproate production from L-leucine
Source: PLoS One. 2017 Jun 29;12(6):e0179229. doi: 10.1371/journal.pone.0179229 (PMC5491005; doi:10.1371/journal.pone.0179229)
Supplement: S3 Table — (DOCX) [file pone.0179229.s004.docx]

**S3 Table. Primers used in the study.**

| **Primers Sequences (5’-3’)** | |  | | | |  |
| --- | --- | --- | --- | --- | --- | --- |
| **Primers for optimization of plasmid copy number** | | |  | | |  |
| LADf | **CATGCCATGG**CGATATCTAGAAGAAAATTTA | | | *Nco* I |  |  |
| LADd | **CCGGAGCTC**TTAGAATCTGTAAAGACTAAATGGTTT | | | *Xho* I |  |  |
| **Primers for constructing mutants at DR** | |  | | | |  |
| A2-F | CTTTAAGAAGGAGATATACCATGGCTATATCTAGAAGAAA | | |  |  |  |
| A2-D | TTTCTTCTAGATATAGCCATGGTATATCTCCTTCTTAAAG | | |  |  |  |
| S4-F | CCATGGCGATATCAAGAAGAAAATTTATCATTGGTGG | | |  |  |  |
| S4-D | CCACCAATGATAAATTTTCTTCTTGATATCGCCATGG | | |  |  |  |
| R5-F | CATGGCGATATCTAGGAGAAAATTTATCATTGGTGG | | |  |  |  |
| R5-D | CCACCAATGATAAATTTTCTCCTAGATATCGCCATG | | |  |  |  |
| R6-F | CATGGCGATATCTAGAAGGAAATTTATCATTGGTGG | | |  |  |  |
| R6-D | CCACCAATGATAAATTTCCTTCTAGATATCGCCATG | | |  |  |  |
| I9-F | CTAGAAGAAAATTTATAATTGGTGGAACAGTGGTGGC | | |  |  |  |
| I9-D | GCCACCACTGTTCCACCAATTATAAATTTTCTTCTAG | | |  |  |  |
| I10-F | CTAGAAGAAAATTTATAATTGGTGGAACAGTGGTGGC | | |  |  |  |
| I10-D | GCCACCACTGTTCCACCAATTATAAATTTTCTTCTAG | | |  |  |  |
| G11-F | TTTATCATTGGAGGAACAGTGGTGGCTGTC | | |  |  |  |
| G11-D | GACAGCCACCACTGTTCCTCCAATGATAAA | | |  |  |  |
| I3Δ-F | GAGATATACCATGGCGTCTAGAAGAAA | | |  |  |  |
| I3Δ-D | TTTCTTCTAGACGCCATGGTATATCTC | | |  |  |  |
| S4Δ-F | CCATGGCGATAAGAAGAAAATTTATCATTGG | | |  |  |  |
| S4Δ-D | CCAATGATAAATTTTCTTCTTATCGCCATGG | | |  |  |  |
| R5Δ-F | GGCGATATCTAGAAAATTTATCATTGGTGG | | |  |  |  |
| R5Δ-D | CCACCAATGATAAATTTTCTAGATATCGCC | | |  |  |  |
| K7Δ-F | GGCGATATCTAGAAGATTTATCATTGGTGG | | |  |  |  |
| K7Δ-D | CCACCAATGATAAATCTTCTAGATATCGCC | | |  |  |  |
| F8Δ-F | CTAGAAGAAAAATCATTGGTGGAACAGTGG | | |  |  |  |
| F8Δ-D | CCACTGTTCCACCAATGATTTTTCTTCTAG | | |  |  |  |
| **Primers for optimization of RBS** | |  | | | |  |
| RBS-1 | TACGGTATATACCATGGCGATATCTAGAAGAA | | |  |  |  |
| RBS-2 | TGCGGTATATACCATGGCGATATCTAGAAGAA | | |  |  |  |
| RBS-3 | CTGCGGATATACCATGGCGATATCTAGAAGAA | | |  |  |  |
| RBS-4 | CTACGGATATACCATGGCGATATCTAGAAGAA | | |  |  |  |
| RBS-5 | GCGGTAATATACCATGGCGATATCTAGAAGAA | | |  |  |  |
| RBS-6 | CGGTTAATATACCATGGCGATATCTAGAAGAA | | |  |  |  |
| RBS-7 | TACGGAATATACCATGGCGATATCTAGAAGAA | | |  |  |  |
| RBS-8 | TATGGTATATACCATGGCGATATCTAGAAGAA | | |  |  |  |
| RBS-9 | GCGGCGATATACCATGGCGATATCTAGAAGAA | | |  |  |  |
| RBS-10 | TCGGTCATATACCATGGCGATATCTAGAAGAA | | |  |  |  |
| RBS-11 | GGGCGGATATACCATGGCGATATCTAGAAGAA | | |  |  |  |
| RBS-12 | ACGGGTATATACCATGGCGATATCTAGAAGAA | | |  |  |  |
| RBS-13 | GCCCGGATATACCATGGCGATATCTAGAAGAA | | |  |  |  |
| RBS-14 | CCCGGCATATACCATGGCGATATCTAGAAGAA | | |  |  |  |
| RBS-15 | CTATGTATATACCATGGCGATATCTAGAAGAA | | |  |  |  |
| RBS-16 | CTTGGTATATACCATGGCGATATCTAGAAGAA | | |  |  |  |
| RBS-17 | GTAGTAATATACCATGGCGATATCTAGAAGAA | | |  |  |  |
| RBS-18 | TGGTAGATATACCATGGCGATATCTAGAAGAA | | |  |  |  |
| **RBS-19**  **(wild-type)** | **AAGGAGATATACCATGGCGATATCTAGAAGAA** | | |  |  |  |
| RBS-20 | CTCGACATATACCATGGCGATATCTAGAAGAA | | |  |  |  |
| RBS-21 | ACTTCTATATACCATGGCGATATCTAGAAGAA | | |  |  |  |
| RBS-22 | CCGAGGATATACCATGGCGATATCTAGAAGAA | | |  |  |  |
| RBS-23 | CACGCCATATACCATGGCGATATCTAGAAGAA | | |  |  |  |
| RBS-24 | CCATGTATATACCATGGCGATATCTAGAAGAA | | |  |  |  |
| RBS-25 | TTCTAGATATACCATGGCGATATCTAGAAGAA | | |  |  |  |
| RBS-26 | AGAGCCATATACCATGGCGATATCTAGAAGAA | | |  |  |  |
| RBS-27 | ATAGCCATATACCATGGCGATATCTAGAAGAA | | |  |  |  |
| RBS-rev | CTTAAAGTTAAACAAAATTATTTCTAGAGG | | |  |  |  |
| **Primers for qRT-PCR** | |  | | | |  |
| LAD-F | GCTGATGTTGTTGTTGTT | | |  | | |
| LAD-D | TTGCCTGACCATAGAATC | | |  | | |
| 16S rRNA-F | TAATACCTTTGCTCATTG | | |  | | |
| 16S rRNA-D | CCAGTAATTCCGATTAAC | | |  | | |
